# An innovative lab-scale production for a novel therapeutic DNA vaccine candidate against rheumatoid arthritis

**DOI:** 10.1186/s13036-024-00411-w

**Published:** 2024-02-27

**Authors:** Juan Long, Xiao Zhao, Fei Liang, Yang Zeng, Nan Liu, Yuying Sun, Yongzhi Xi

**Affiliations:** 1 National Key Laboratory of Blood Science, Senior Department of Hematology, Fifth Medical Center of Chinese PLA General Hospital, No. 8, Dongda Ave, Fengtai District, Beijing, 100071 China; 2 Forregen (Beijing) Bioscience-Technology Development Centre Co., Ltd, Qingquan Villa Yili of Beijing Fragrant Hill, Haidian District, Beijing, 100093 China

**Keywords:** Therapeutic-plasmid DNA vaccine, PcDNA-CCOL2A1 vaccine, Lab-scale plasmid DNA purification, PEG/MgCl_2_ precipitation, Triton X-114

## Abstract

**Background:**

Recent therapeutic-plasmid DNA vaccine strategies for rheumatoid arthritis (RA) have significantly improved. Our pcDNA-CCOL2A1 vaccine is the most prominent and the first antigen-specific tolerising DNA vaccine with potent therapeutic and prophylactic effects compared with methotrexate (MTX), the current “gold standard” treatment for collagen-induced arthritis (CIA). This study developed a highly efficient, cost-effective, and easy-to-operate system for the lab-scale production of endotoxin-free supercoiled plasmids with high quality and high yield. Based on optimised fermentation culture, we obtained a high yield of pcDNA-CCOL2A1 vaccine by PEG/MgCl_2_ precipitation and TRION-114. We then established a method for quality control of the pcDNA-CCOL2A1 vaccine. Collagen-induced arthritis (CIA) model rats were subjected to intramuscular injection of the pcDNA-CCOL2A1 vaccine (300 μg/kg) to test its biological activity.

**Results:**

An average yield of 11.81 ± 1.03 mg purified supercoiled plasmid was obtained from 1 L of fermentation broth at 670.6 ± 57.42 mg/L, which was significantly higher than that obtained using anion exchange column chromatography and a commercial purification kit. Our supercoiled plasmid had high purity, biological activity, and yield, conforming to the international guidelines for DNA vaccines.

**Conclusion:**

The proposed innovative downstream process for the pcDNA-CCOL2A1 vaccine can not only provide a large-scale high-quality supercoiled plasmid DNA for preclinical research but also facilitate further pilot-scale and even industrial-scale production of pcDNA-CCOL2A1 vaccine.

## Background

In recent years, substantial progress has been made in the development of tolerating DNA vaccines as a novel strategy for the treatment of rheumatoid arthritis (RA) [1–4]. Among these major advances, our therapeutic pcDNA-CCOL2A1 vaccine is the most prominent because it is the first antigen-specific tolerising DNA vaccine encoding chicken type II collagen with a 4837 bp full-length cDNA [5]. Notably, a series of recent studies have demonstrated that the pcDNA-CCOL2A1 vaccine has potent therapeutic and prophylactic effects compared with those of methotrexate (MTX), the current “gold standard” treatment for collagen-induced arthritis (CIA) in rats [6, 7]. Furthermore, intramuscular injection vaccination with the pcDNA-CCOL2A1 vaccine can induce better specific humoral and cellular immune responses than subcutaneous and intravenous injection vaccination against CIA. A single subcutaneous or intramuscular injection with the pcDNA-CCOL2A1 vaccine can maintain the curative effect for over a month, greatly improving drug compliance [8]. In addition, the pcDNA-CCOL2A1 vaccine was confirmed to be safe, non-immunogenic, and well-tolerated, with no detectable adverse clinical events [9]. More importantly, no exogenous *CCOL2A1* gene was integrated into the host genome after inoculation [10]. These results strongly indicate the high drug-ability of the pcDNA-CCOL2A vaccine, which inspired us to further develop efficient downstream process technologies of the DNA vaccine for preclinical research and clinical applications.

Recently, an increasing number of DNA vaccines and gene therapies have entered the preclinical research and clinical application stages, respectively [11, 12]. However, for DNA vaccines or gene therapies to be formally approved for preclinical research and clinical applications, obtaining sufficient high-purity and high-quality supercoiled plasmid DNA is essential. Hence, the US Food and Drug Administration (FDA, USA), European Medicines Evaluation Agency (EAEM, Europe), and National Medical Products Administration (NMPA, China) have issued regulatory documents related to the preparation of pharmaceutical-grade plasmid DNA. These documents describe the entire production process, including the selection of cell lines, raw materials, purification, identification, and final production and marketing [13–15]. In addition, to ensure safety, residual linear and denatured plasmids, genomes, endotoxins, and other impurities must be removed to a maximum extent from the purified final products of supercoiled plasmid DNA. Thus, establishing simple, efficient, economical, easily controlled, high-quality, widely applicable, and easy-to-scale separation and purification methods for sufficient supercoiled plasmid DNA has become one of the biggest challenges in this field today, although classical, commonly used, and commercially available purification kits can meet the needs of small-scale academic research in the laboratory.

Moreover, owing to differences in the target genes, expression vectors, and host bacteria of each genetic engineering product, their downstream process technologies are also different. No mature, standardised, and universal downstream process technologies have been developed for therapeutic DNA vaccines. This has become a major obstacle restricting the clinical application of DNA vaccines [16]. In this study, we attempted to develop and optimise a high-efficiency, cost-effective, easy-to-operate system for lab-scale separation and purification of the pcDNA-CCOL2A1 vaccine, which will not only provide a large-scale yield of high-quality vaccine products for preclinical research but also facilitate further pilot-scale and industrial-scale production of the pcDNA-CCOL2A1 vaccine.

## Material and methods

### Plasmid and bacterial strains

The eukaryotic expression vector pcDNA3.1( +)-CCOL2A1 containing the exogenously expressed *CCOL2A1* gene was successfully constructed in our laboratory [5, 17]. The recombinant plasmid was cloned in *E. coli* DH5α cells (CB101; Tiangen, Beijing, China).

### Fermentation

The fermentation processes were carried out according to our optimised conditions, as previously described [18]. The final product pcDNA-CCOL2A1 was quantified using a Synergy HT Multi-Mode microplate reader (BioTek Instruments, Inc., Winooski, VT, USA).

### Alkaline lysis

The bacterial pellet obtained from a 100-mL flask of fermentation broth was suspended in 10 mL suspension buffer (25 mM Tris/HCl, 10 mM EDTA, 50 mM glucose, pH 8.0). The bacterial suspension was then mixed with 20 mL lysis solution (0.2 N NaOH, 1.0% SDS) and incubated at 25 °C for 7 min, as described by Sambrook and Rusell [19]. The resulting lysate was neutralised with 20 mL neutralisation buffer (3 M KAc, pH 5.5) and incubated on ice for 10 min. The turbid lysate was centrifuged at 4 °C for 30 min at 12,000 × *g*. Cellular debris, genomic DNA, and most of the host proteins were removed from the bacterial lysis fluid via centrifugation, and the supernatant was immediately transferred to a fresh vessel for further extraction and purification [20].

### Plasmid DNA purification

**Fig. 1:**
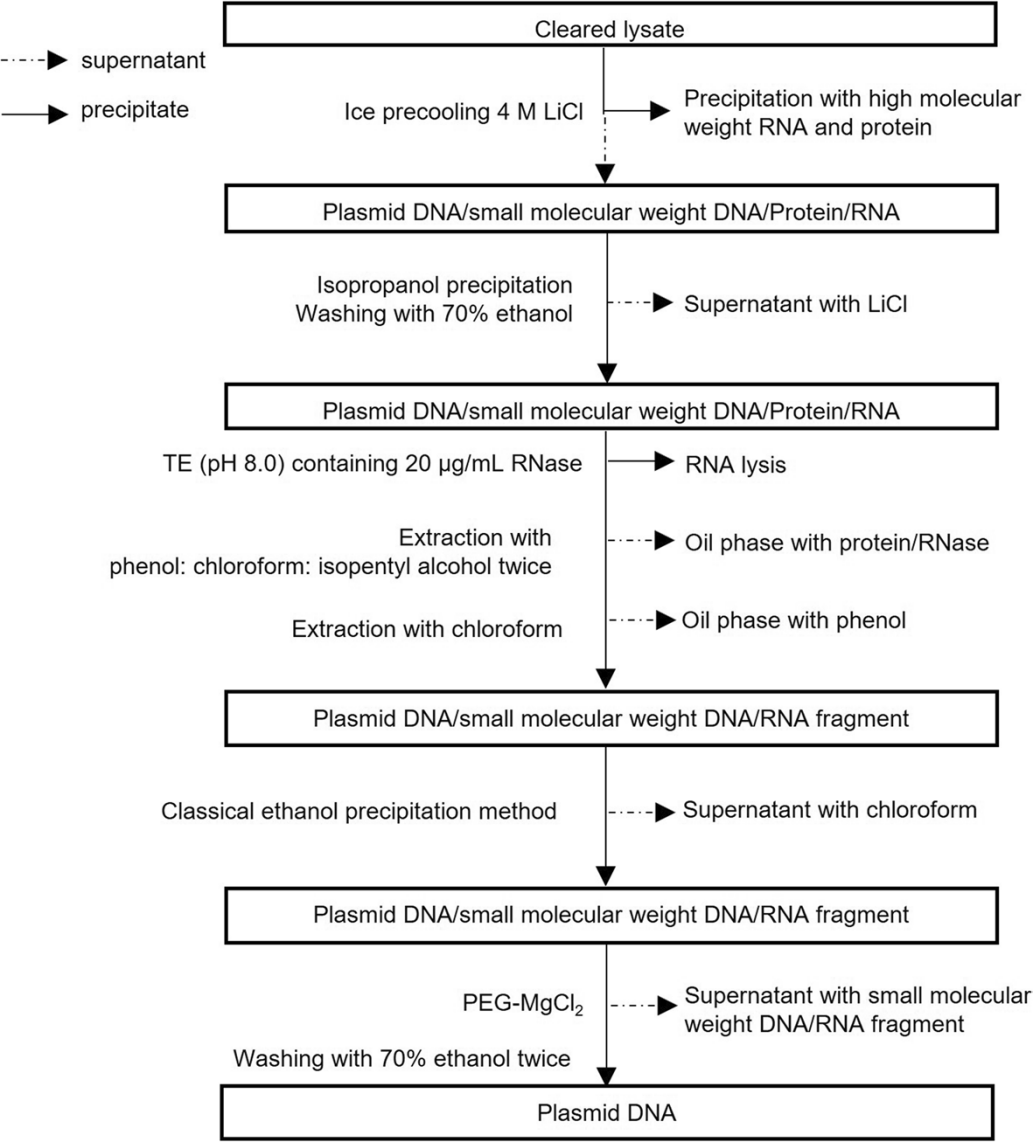
Process illustration and overview of the PEG/MgCl_2_ precipitation protocol. The full arrow represents the pellet fraction, and the dashed arrow represents the supernatant fraction after centrifugation at 12,000 × *g*. All precipitations were performed at 25 °C

LiCl is widely used to precipitate high-molecular-weight RNA and proteins [19]. An equal volume of precooled LiCl (4 M) was added to the cleared lysates, which were collected from the alkaline lysis process. The resulting supernatant was precipitated using isopropyl alcohol and washed with 70% ethanol to elute the LiCl. The RNase [recombinant RNase, Sangon Biotech (Shanghai) Co., Ltd., China] and protein fragments produced during cell lysis were removed by performing phenol extraction twice. The standard ethanol precipitation method was subsequently used to remove the organic reagents introduced during extraction. Plasmid DNA was then precipitated using polyethylene glycol (PEG)/MgCl_2_ (20% PEG-8000, 15 mM MgCl_2_) to elute small molecular DNA and RNA fragments (Fig. 1).

### Endotoxin removal

**Fig. 2:**
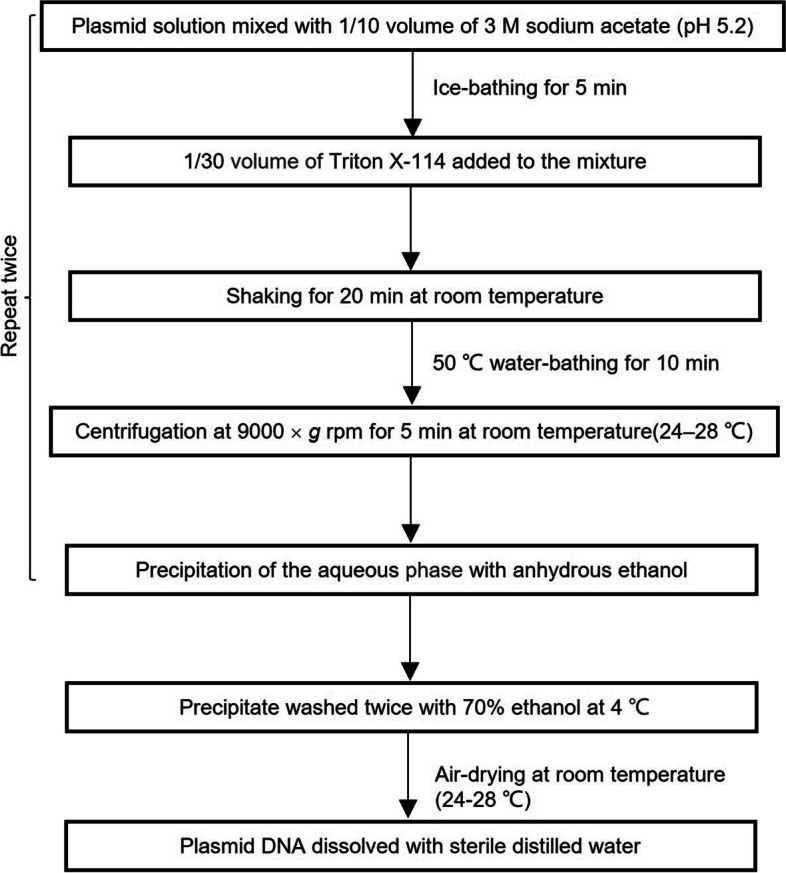
Schematic outline of the Triton X-114 method in eliminating endotoxins from the pcDNA-CCOL2A1 vaccine

Triton X-114 phase separation is a simple and cost-effective strategy for eliminating endotoxins from plasmid DNA [21, 22]. Repeated validation experiments showed that the endotoxin residues met the requirements set forth by NMPA, EAEM, and FDA (≤ 10 EU/mg as per the NMPA and EAEM, ≤ 40 EU/mg as per the US FDA) [13–15] (Fig. 2).

### Anion exchange chromatography (AEC)

Before AEC, the endotoxins in the plasmid were removed using Triton X-114. The endotoxin-free bacterial lysate was processed using a Qiagen Anion-Exchange Resin packed in a chromatography column to a final bed volume of 50 mL and equilibrated with equilibration buffer (750 mM NaCl; 50 mM MOPS, pH 7.0; 15% isopropanol; 0.15% Triton X-100) at a flow rate of 15–20 mL/min. The plasmid samples were then loaded at a flow rate of 15–20 mL/min. The chromatography column was washed with wash buffer (40 mL; 1.0 M NaCl; 50 mM MOPS, pH 7.0; 15% isopropanol) at a flow rate of 20–30 mL/min, followed by elution of the plasmid DNA with elution buffer (18 mL; 1.6 M NaCl; 50 mM MOPS, pH 7.0; 15% isopropanol) at a flow rate of 8–10 mL/min. The eluted plasmid DNA solution was immediately transferred to a new vessel and mixed with isopropanol (0.7 volumes). The DNA pellet was washed with endotoxin-free 70% ethanol, air-dried for approximately 20 min, and re-dissolved with endotoxin-free water (2 mL).

### Purification using a commercial purification kit

An Endo-Free Plasmid Mega kit (Qiagen, Valencia, CA, USA), which was designed for the purification of endotoxin-free plasmid DNA, was used as the control trial. The assay was conducted following the manufacturer’s plasmid purification protocols.

### Concentration and purity analysis

The final plasmid DNA was quantified using a multi-mode microplate reader at a wavelength of 260 nm. The total DNA concentration was calculated using the formula: A_260_ × dilution factor × 50 μg/mL.

### Endotoxin analysis

Endotoxin contamination was assessed using a Limulus Amebocyte Lysate (LAL) Assay kit (Xiamen Bio-endo Technology, Co., Ltd., China) according to the manufacturer’s instructions. The A_405_ of the samples was detected using the multi-mode microplate reader. The detection level of this assay kit was 0.1 EU/mL.

### *Detection of *E. coli* proteins*

The concentrations of *E. coli* proteins in the final plasmid product were determined using a Micro *E. coli* DH5α Protein Assay Reagent kit (WENZHOU Kemiao Biotechnology, Co., Ltd., China), according to the manufacturer’s protocol. The absorbance of the reaction mixture was measured at 450 nm using the multi-mode microplate reader. The detection range of this assay kit was 0.8–24 ng/L.

### *Detection of* E. coli *genomic DNA*

*E. coli* genomic DNA was detected using quantitative real-time polymerase chain reaction (qPCR) under the following parameters; each 20-μL reaction system contained 10 μL of 2 × T5 Fast qPCR Mix (SYBR Green I), 1 μL of 10 μM forward/reverse primers, and 1 μL gDNA template. Thermal cycling was performed using an FQD-96A Sequence Detection System (BIOER Technology, Hangz, China) with the following thermocycling parameters: inactivation of reverse transcriptase at 95 °C for 5 min, then 40 cycles of 95 °C for 15 s, 56 °C for 15 s, and 72 °C for 20 s, with single-point fluorescence detection. The sequences of the *E. coli* 16S rRNA primers used in this study are as follows: forward, 5′-CCGTTTCTCACCGATGAACA-3′; reverse, 5′-GCTGTCGATGACAGGTTGTT-3′.

### Detection of residual RNA

Residual RNA was detected using horizontal electrophoresis on 0.8% agarose gels (5 V/cm, 25 min). The gels were analysed using ImageJ (Laboratory for Optical and Computational Instrumentation, LOCI, University of Wisconsin).

### Supercoiled DNA analysis

Superhelical structure evaluation of the plasmid DNA was performed using high-performance liquid chromatography (HPLC; Alliance 2795; Waters, USA) on an Ultimate AQ-C18 column (Waters, USA) with the mobile phase A as 0.1 M TEAA (pH 7.0) and mobile phase B as acetonitrile. The column was first equilibrated with 2 CV of phase A. Following sample injection (300 μL), linear gradient elution was performed with the following elution conditions: the initial ratio of phase A: phase B was 100%:0%, elution was for 25 min, and the final ratio of phase A: phase B was 2%:98%.

### Sequencing analysis

The final plasmid DNA purified using the three purification processes was subjected to sequencing and restriction digestion analyses. Sequencing was conducted, and the results were compared with the reported *CCOL2A1* sequence using the DNAMAN v5.2.2 software (Lynnon Biosoft Corp., USA). The purified plasmid DNA was linearised with EcoRI and HindIII (TaKaRa Bio, Inc., Shiga, Japan).

### Detection of biological activity

Six-week-old inbred female Wistar rats (the Animal Breeding Centre of the Academy of Military Medical Sciences, Beijing, China) were randomly divided into six groups (10 animals per group). The inoculation groups were injected with the pcDNA-CCOL2A1 vaccine intramuscularly (300 μg/kg) in the left hind limb. The positive control group received an intramuscular injection of MTX (0.75 mg/kg) once a week for 4 weeks, while the negative control received a single intramuscular injection of normal saline (NS) [7]. Fourteen days after vaccination with the DNA plasmid, a CIA model was induced and evaluated as described previously [7, 8]. All the animal experiments were carried out in accordance with the National Research Council's Guide for the Care and Use of Laboratory Animals.

### Statistical analysis

For descriptive analyses, data are presented as means ± SD. Analysis of variance was performed to evaluate the significance level between the experiment and control groups using the SPSS13.0 software. If the test of homogeneity of variance showed no homogeneity of variance between groups (*P* > 0.05), the least significant difference test was used for multiple comparisons between groups. Otherwise, Tamhane analysis was performed.

## Results

### Yield of the therapeutic pcDNA-CCOL2A1 vaccine

An overview of the plasmid DNA yield is presented in Table 1. The plasmid yield was described using three parameters: concentration, volumetric plasmid yield (the weight of the plasmid DNA per litre of fermentation broth), and special plasmid yield (the weight of the plasmid DNA per gram wet cell weight). The volumetric plasmid yield obtained from the combination procedure of PEG/MgCl_2_ precipitation and Triton X-114 was 11.81 ± 1.03 mg/L, significantly higher than that obtained using AEC and a commercial kit (*P* = 0.008 and 0.032, respectively). The volumetric plasmid yield obtained using AEC was 6.62 ± 0.79 mg/L, which was not significantly different from that obtained using a commercial kit (6.52 ± 0.15 mg/L;* P* = 0.997). The special plasmid yield obtained using the combination procedure was 1.78 ± 0.30 mg/g, almost twice that obtained using AEC (0.83 ± 0.05 mg/g, *P* = 0.001) and the commercial extraction kit (0.77 ± 0.11 mg/g, *P* = 0.001). The concentrations of the plasmid DNA purified using the combination procedure were also significantly higher than those from the other two processes used in this study (Table 1). The final plasmid concentration in the PEG/MgCl_2_ group was 670.60 ± 57.42 mg/L, which was also twice that obtained using AEC and a commercial purification kit, at 330.81 ± 39.61 and 325.75 ± 7.67 mg/L, respectively.

**Table 1:** Yield of the pcDNA-CCOL2A1 vaccine following different purification procedures

Purification method	Volumetric plasmid yield (mg/L)	Specific plasmid yield (mg/g)	Concentration (mg/L)
PEG/MgCl_2_	11.81 ± 1.03*	1.78 ± 0.30*	670.60 ± 57.42**
AEC	6.62 ± 0.79	0.83 ± 0.05	330.81 ± 39.61
Commercial kit	6.52 ± 0.15	0.77 ± 0.11	325.75 ± 7.67

Data are expressed as the means ± standard deviations of three independent experiments

*P < 0.05, **P < 0.001

Abbreviations: *AEC* Anion exchange column chromatography, *PEG/MgCl*_*2*_ Polyethylene glycol/magnesium chloride

### Quality and purity of the therapeutic pcDNA-CCOL2A1 vaccine

Purifying plasmid DNA vaccines for therapeutic applications and animal trials essentially aims to eliminate host residues, such as endotoxins, proteins, genomic DNA, and RNA. To reduce side reactions and ensure the reproducibility of plasmid DNA vaccine activity, the impurities in plasmid DNA vaccine should meet certain acceptance criteria [13–15]. Table 2 provides information on the impurities, analytical methods, testing results, and acceptance criteria. The LAL assay showed that the amount of endotoxin residues complied with the FDA, NMPA, and EAEM acceptance criteria, regardless of the method used. Contaminants, such as *E. coli*, were not detected using enzyme-linked immunosorbent assay (ELISA), meaning its content was far below the detection limit of 1 μg/mg [13–15]. The amounts of *E. coli* genomic DNA in the plasmid DNA purified using the PEG/MgCl_2_ precipitation protocol and AEC were 2.09 ± 0.18 μg/mg and 3.45 ± 0.57 μg/mg, respectively, both of which were significantly lower than the 7.49 ± 0.07 μg/mg plasmid DNA obtained using the commercial kit (*P* = 0.000 and 0.018, respectively) [13–15]. All residues of *E. coli* genomic DNA in the final products obtained using these three methods complied with the EAEM guidelines [14].

**Table 2:** Quality analysis of the pcDNA-CCOL2A1 vaccine via different purification procedures

Impurity	Analytical method	PEG/MgCl_2_	IEC	Commercial kit	Acceptance criteria
Appearance	Visual inspection	Clear, colourless			Clear, colourless
A_260_/A_280_	UV spectrophotometer	1.98 ± 0.02	2.00 ± 0	2.10 ± 0.10	≥ 1.75^a^ 1.75–1.85^b^
Endotoxin (EU/mg)	LAL assay	4.25 ± 0.40	5.31 ± 0.53	4.66 ± 0.48	≤ 10 EU/mg^a,^ ^b^ ≤ 40 EU/mg ^c^
*E. coli* protein (μg/mg)	ELISA	Undetectable			≤ 1 μg/mg^a^ ≤ 10 μg/mg ^b^ < 1% ^c^
*E. coli* gDNA (μg/mg)	Q-PCR	2.09 ± 0.18**	3.45 ± 0.57*	7.49 ± 0.07	≤ 2 μg /mg^a^ ≤ 10 μg/mg ^b^ < 1%^c^
*E. coli* RNA (%)	AGE	Not visible	10.11%	Not visible	Not visible^a,^ ^b^ < 1%^c^
Supercoiled (%)	HPLC/AGE	94.98%	71.24%	93.67%	≥ 90%^a, b^ ≥ 80%^c^

Data are expressed as the mean ± standard deviation (SD) of three independent batch experiments. *P < 0.05, ** *P* < 0.001

Abbreviations:* AGE* Agarose gel electrophoresis, *ELISA *Enzyme-linked immunosorbent assay, *EU/mg *Endotoxin units per milligram, *gDNA *genomic DNA, *HPLC *High-performance liquid chromatography, *LAL assay *Limulus amoebocyte lysate assay, *Q-PCR *Quantitative polymerase chain reaction

^a^NMPA guidelines

^b^EAEM guidelines

^c^US FDA guidelines

A gel imaging scanning analyser was used to quantify the RNA residues in the plasmid product. The plasmid DNA obtained using PEG/MgCl_2_ precipitation and the commercial kit had no visible RNA bands, whereas the plasmids obtained using the AEC method had obvious RNA bands, with a percentage of 10.11%. This is because both RNA and plasmid DNA are nucleic acid molecules with negative charges and could not be effectively separated using AEC alone. Therefore, a combination of tangential flow filtration and multi-step chromatography or selective precipitation is commonly used to remove RNA contaminants from plasmid DNA [20, 23, 24]. Our results confirmed that PEG/MgCl_2_ precipitation could effectively precipitate small-molecule RNA from the pcDNA-CCOL2A1 vaccine.

### Homogeneity analysis of the therapeutic pcDNA-CCOL2A1 vaccine

**Fig. 3:**
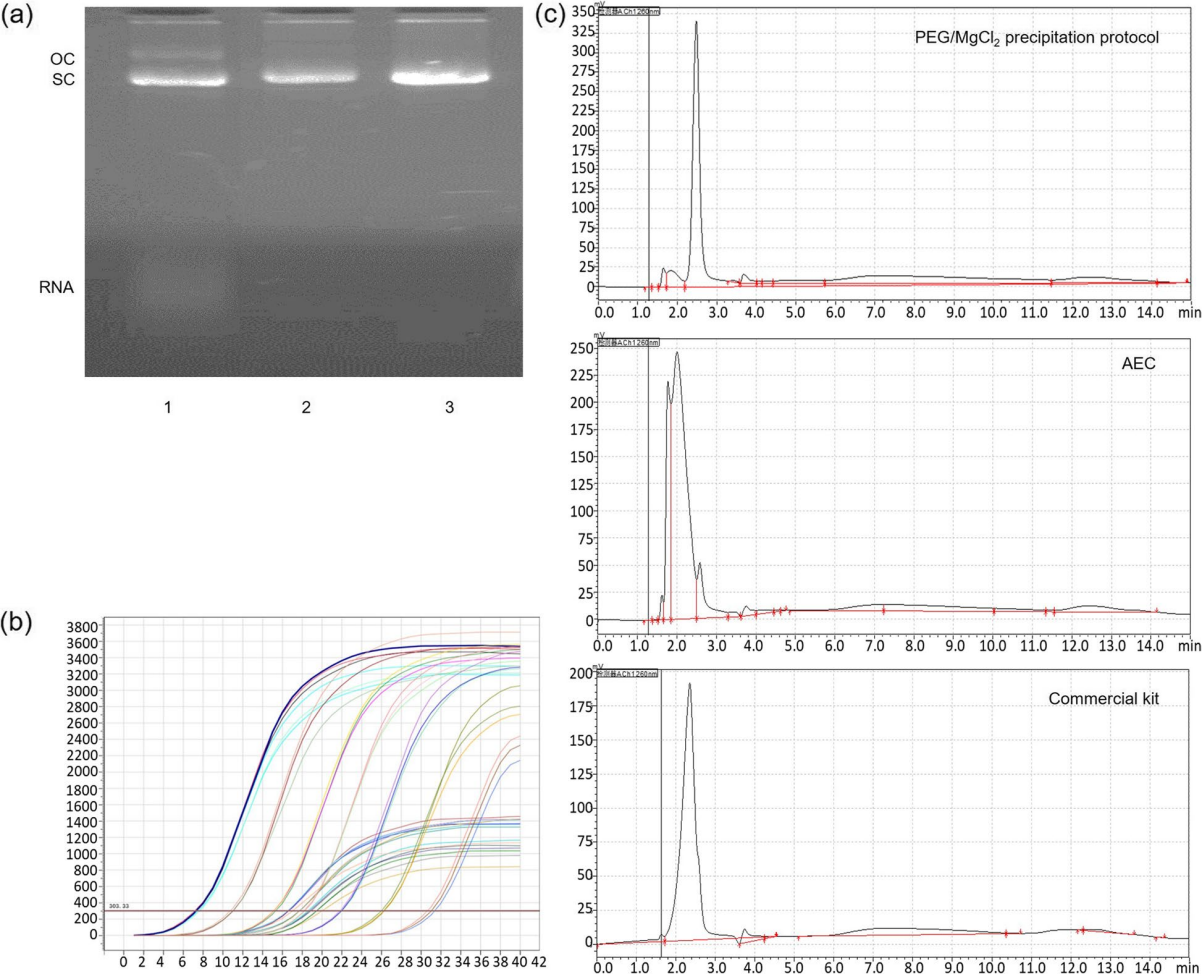
Purity analysis of the final plasmid products. **a** Analysis of the final plasmid using 0.8% agarose gel electrophoresis (5 V/cm, 35 min). Lane 1: plasmid purified via anion exchange column chromatography (AEC). Lane 2: plasmid purified using a commercial kit. Lane 3: plasmid purified using a combination of PEG/MgCl_2_ precipitation and Triton X-114. OC: open circle; SC: supercoiled circle. **b** The amplification curves include gene-positive samples assessed with three duplicates per sample using real-time quantitative PCR (RT-qPCR). **c** Analysis of plasmid forms after purifying the pcDNA-CCOL2A1 vaccine via high-performance liquid chromatography (HPLC)

Supercoiled DNA is the active isoform of plasmid DNA for expression, translation, and eliciting immune responses in vivo; it directly affects the biological activity of gene products. Other isoforms of plasmid DNA, such as open circular, linear, and denatured plasmid DNA, are commonly generated during fermentation and purification. Furthermore, the content of supercoiled DNA in the final product is related to the host strains and plasmid [25]. The FDA recommends a supercoiled plasmid content of ≥ 80%, whereas the NMPA and EAEM recommend more than 90% [13–15]. In this study, supercoiled DNA analysis of the final product was performed via agarose gel electrophoresis and HPLC. The results demonstrated a high percentage of supercoiled plasmid purified using PEG/MgCl_2_ purification and the commercial kit, with supercoiled DNA percentages of 94.98% and 93.67%, respectively (Fig. 3). However, the amount of supercoiled plasmid obtained using AEC was 71.24%, indicating that different isomers of the pcDNA-CCOL2A1 vaccine could not be effectively separated using AEC alone.

### Molecular characterisation of the therapeutic pcDNA-CCOL2A1 vaccine

**Fig. 4:**
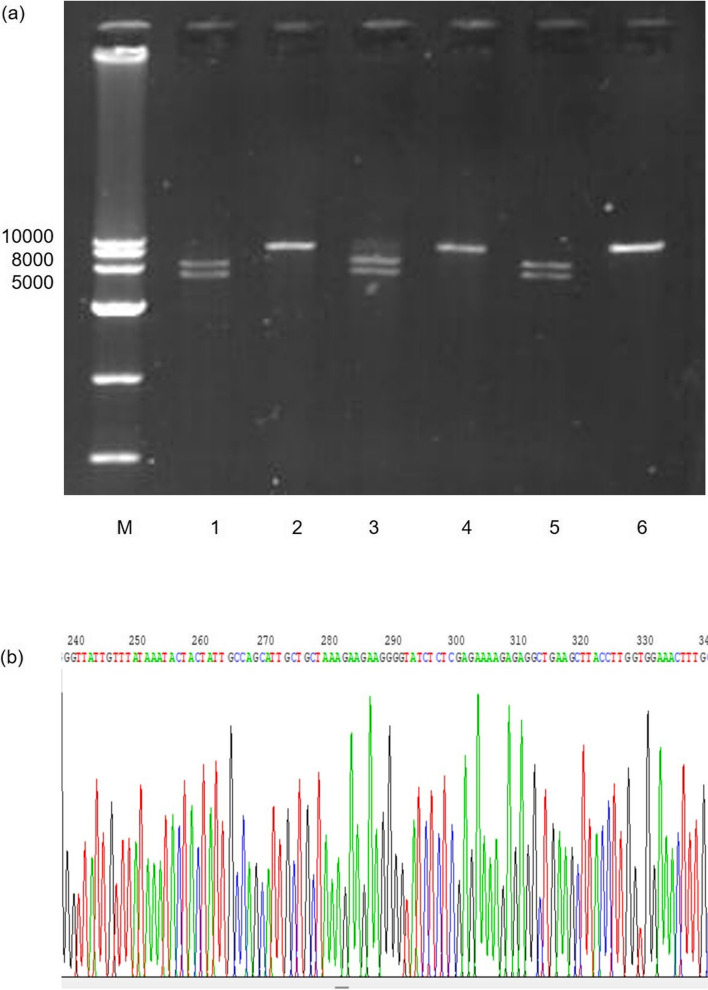
Identification of the final plasmid products. **a** The final plasmid extracted using the different processes was subjected to restriction enzyme digestion and analysed using 1% agarose gel electrophoresis (5 V/cm, 45 min). M: DNA Marker (1 kb Plus DNA Marker, MD113, TIANGEN). Lanes 1 and 2: plasmids purified using the PEG/MgCl_2_ precipitation protocol linearised with HindIII and double-digested with HindIII and EcoRI, respectively. Lanes 3 and 4: plasmids purified using anion exchange chromatography (AEC) linearised with HindIII and double-digested with HindIII and EcoRI, respectively. Lanes 5 and 6: plasmids purified using a commercial kit linearised with HindIII and double-digested with HindIII and EcoRI, respectively. **b** Extract of a typical chromatogram obtained from sequencing the plasmids obtained in this study

Regardless of the purification process, all the obtained plasmid samples were suitable for downstream applications, such as sequencing and enzyme digestion (Fig. 4).

### Biological activity of the therapeutic pcDNA-CCOL2A1 vaccine

**Fig. 5:**
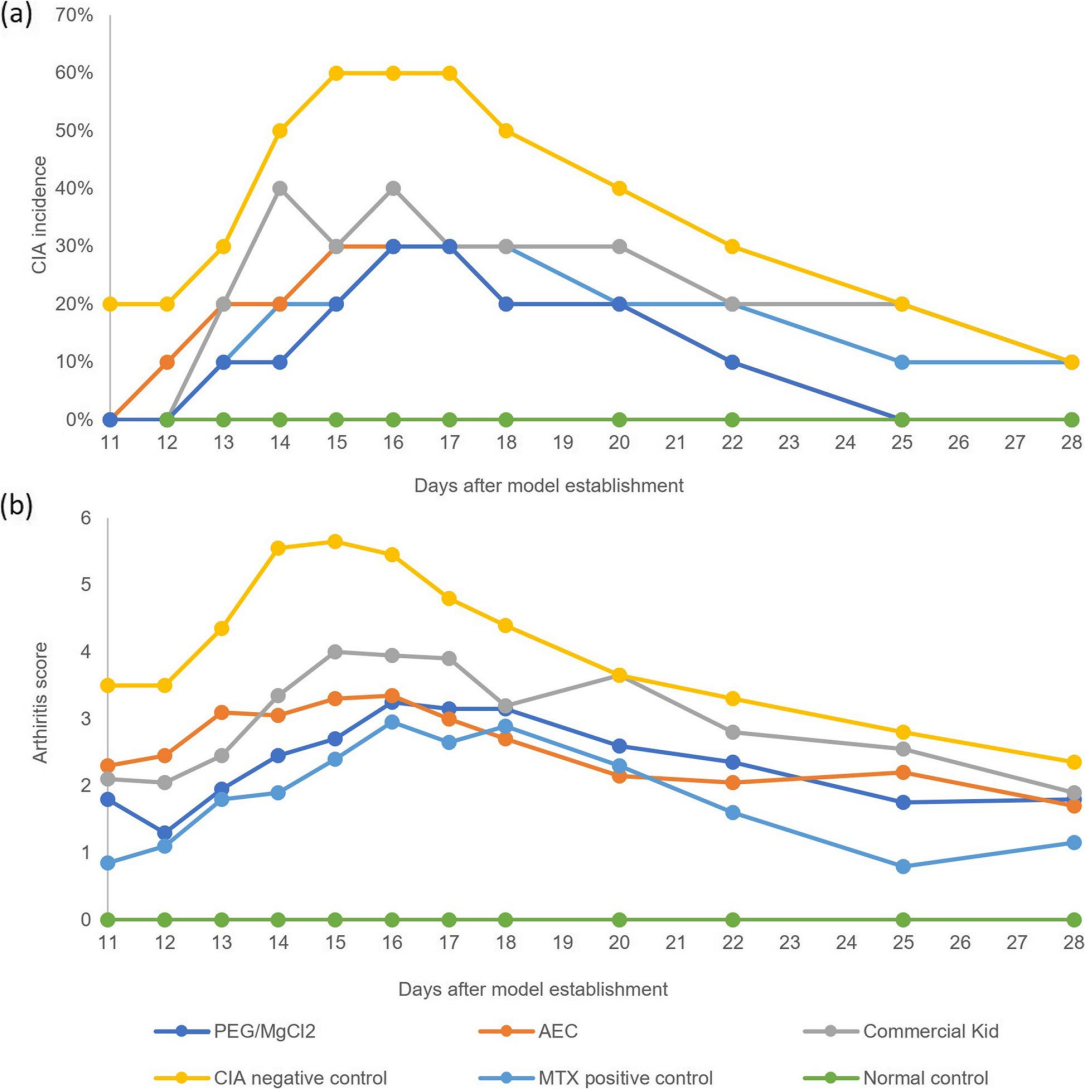
Assessment of the biological efficacy of the plasmid DNA vaccine on collagen-induced arthritis (CIA) rats. **a** The incidence of arthritis in the experimental rats during the experimental period. **b** The arthritis score of the experimental rats during the experimental period. The plasmid DNA vaccine was administered on day 14 before the onset of arthritis, and arthritis progression was monitored over 28 days using a macroscopic scoring system. The data represent the average gross clinical scores for rats treated with 300 μg/kg of vaccine, normal saline (NS), and methotrexate (MTX) (*n* = 10 for each group). Error bars have been omitted for clarity. These data are representative of three independent experiments, which yielded similar results

The bioactivity of the pcDNA-CCOL2A1 plasmid produced from the three different purification processes was evaluated, as reported previously [7, 8]. The results showed that the plasmid product obtained through the different purification methods could significantly reduce the incidence and severity of CIA in rat models, consistent with our previous investigation [7]. Additionally, the physical characteristics of the experimental rats remained normal during the entire observation period. A detailed demonstration of the biological activity of the plasmid is shown in Fig. 5.

## Discussion

In this study, we innovatively developed a high-efficiency, cost-effective, and easy-to-operate system for the lab-scale separation and purification of the pcDNA-CCOL2A1 vaccine. Furthermore, we confirmed that the residual *E. coli* protein, genome, RNA, and endotoxins in the final supercoiled pcDNA-CCOL2A1 vaccine product completely conformed to the international criteria for DNA vaccines [13–15]. Our results will not only provide sufficient high-quality and high-yield pcDNA-CCOL2A1 vaccine for preclinical research but also promote further pilot-scale and even industrial-scale production of pcDNA-CCOL2A1 vaccine. Notably, some important advances worthy of in-depth discussion emerged from this study.

The production of genetic engineering products requires a complex biological engineering system that includes upstream and downstream process technologies. After successfully optimising the upstream process technologies, the downstream process technologies are more critical to the industrialisation of genetic engineering products. Downstream process technologies in genetic engineering typically include large-scale cultures of engineering bacteria (cells) and the separation, purification, and identification of expression products that meet clinical use standards [12]. In recovering genetically engineered products, we should not only pay attention to the use of highly selective separation and purification methods but also consider the reasons that affect the biological activity of the final product and the low utilisation rate of the culture medium during fermentation. Thus, several separation and purification steps are necessary, making downstream process technologies more complex than upstream process technologies. Because a large amount of the pcDNA-CCOL2A vaccine is required for efficacy studies, safety analyses, pharmacokinetic research, and vaccine stability investigations in preclinical trials, we have successfully established a three-tier cell bank and demonstrated the genetic stability of the engineered *E. coli* DH5α carrying the pcDNA-CCOL2A1 plasmid to produce this DNA vaccine with high potential [26]. Furthermore, we have systematically optimised the fermentation process for the engineered *E. coli* strain and greatly increased the yield of plasmid DNA by 51.9%, with the plasmid DNA yield per unit of bacterial liquid reaching 16.97 mg/L [18]. Statistically, the protein content of cell lysates obtained after fermentation is the largest in the unit dry weight, accounting for approximately 55% of the total weight, followed by RNA, which accounts for approximately 21%, and other impurities, such as endotoxins and genomic DNA, which account for approximately 21%; the plasmid DNA only accounts for approximately 3% [27]. Therefore, maximising the yield of plasmid DNA is the first goal of the purification process. In addition, plasmids larger than 10 kb will increase the difficulty of the purification process because larger plasmids are easily affected by shear force. Moreover, obtaining a relatively high proportion of supercoiled plasmid DNA is difficult. Similarly, the yield and purity of the plasmid may also be reduced. Therefore, to facilitate subsequent downstream purification, the pcDNA 3.1( +) expression plasmid, which has a length of only 5.428 kb, was used in our therapeutic pcDNA-CCOL2A1 vaccine [5]. These advances provide a solid foundation for further large-scale separation and purification of the pcDNA-CCOL2A1 vaccine with high quality and yield.

Downstream separation and purification technologies of genetic engineering should meet some requirements. First, the technical conditions should be mild to maintain the biological activity of the target product. Second, the approach should exhibit good selectivity and effectively separate the target product from the complex mixture to achieve a high purification ratio. Additionally, the yield should be high, and the two technologies should connect directly without the need to process or adjust the materials, which can reduce the number of process steps. Finally, the entire separation and purification process should be fast, meeting the requirements of high productivity. Thus, different separation and purification strategies and technical routes are usually formulated for the target plasmid DNA depending on the application, such as lab-scale, pilot-scale, and industrial-scale stages. For example, at the lab-scale stage, purifying plasmid DNA usually involves using a commercial purification kit or the hexadecane trimethylammonium precipitation method. However, these two methods have numerous shortcomings. For example, the quality of the final product is not controllable. Plasmid DNA obtained by different technicians in different batches showed instability and poor reproducibility regarding yield and residual impurities. To purify different plasmid DNA, it is necessary to repeatedly explore the best purification conditions [28]. Additionally, the purification cost is high. The endotoxin removal solution is a patented component of the kit product and cannot be recycled and reused, significantly increasing the purification cost. The chromatogram column used in purification is also non-renewable; thus, the chromatography step greatly increases the purification cost [29, 30]. The approach is also time-consuming, and increasing the reaction to a larger scale is difficult. The use and residual presence of some solvents may also cause safety hazards. Therefore, chromatography techniques are undoubtedly one of the best methods for large-scale plasmid DNA purification, with the advantages of high resolution and high separation efficiency. Commonly used methods include affinity chromatography (AC), size-exclusion chromatography (SEC), and AEC. AC is more sensitive in terms of specificity and selectivity and has therefore become an essential step in the separation of plasmid DNA isomers. SEC is more suitable for the purification of plasmid DNA as a part of downstream purification in combination with other purification methods because the existing media cannot effectively separate the isomers of plasmid DNA. Owing to it versatile functions, AEC can remove a wide range of impurities. However, it is limited by sample volume and quality; therefore, it is suitable for use in the last purification step to achieve the final purification of residual impurities that have not been completely removed in previous purification steps [23, 24]. Overall, the purification of plasmid DNA with different quality requirements can be achieved via chromatography alone or in combination with other methods. Hence, it is imperative to select different chromatographic processes to obtain plasmid DNA that meets the international quality standard. However, two or more chromatography steps will increase costs while decreasing the recovery of plasmid DNA [31].

In our study, following long-term screening and comparison experiments, we optimised and combined the best separation and purification methods, PEG/MgCl_2_ precipitation and Triton X-114. We initially chose a commercial purification kit alone or a chromatography purification method alone, both of which yielded unsatisfactory outcomes. Single AEC not only fails to effectively purify the plasmid DNA but also requires a combination of multi-step chromatography and ultrafiltration in addition to needing a series of high-end instruments. Besides the high costs and yield of plasmid DNA obtained, a single commercial kit also failed to meet the requirements of some preclinical experiments. The cost of producing 1 mg supercoiled plasmid DNA pcDNA-CCOL2A1 using the Qiagen Endo-Free Plasmid Mega kit was US$ 31.67; however, using a combination of PEG/MgCl_2_ precipitation and Triton X-114, the cost was only US$ 1.13. Moreover, by combining PEG/MgCl_2_ precipitation with Triton X-114, an average yield of 11.81 ± 1.03 mg supercoiled plasmid DNA can be purified from 1 L fermentation broth, with a concentration of 670.6 ± 57.42 mg/L, which was significantly higher than that obtained using AEC or the commercial purification kit alone. The plasmid DNA isolated and purified from 1 L fermentation broth could meet the demand of 100 CIA rat models for in vivo efficacy studies, pharmacology assays, and toxicity experiments [8]. In particular, the supercoiled plasmid DNA separated and purified by combining PEG/MgCl_2_ precipitation with Triton X-114 had a high purity and the same biological activity as the plasmid obtained from an internationally used commercial kit. These results indicated that our method was highly efficient, cost-effective, and easy to operate for lab-scale separation and purification of the pcDNA-CCOL2A1 vaccine.

The clinical applications of therapeutic DNA vaccines have broad prospects; however, their safety must be guaranteed before they can be applied to humans. First, quality control mainly considers the purity and consistency of the plasmid DNA vaccine as well as the presence of *E. coli* residual proteins, genome, and endotoxins. Second, the final product must reach a certain purity. The establishment and verification of the quality standard of the purified supercoiled plasmid DNA are essential steps in the overall purification process evaluation and are also the most critical issues for ensuring the safety and effectiveness of subsequent use [11, 12, 16]. Different countries and regions have formulated their quality standards for the quality control of gene products used for therapeutic purposes. Both FDA and EAEM have issued a series of strict evaluation principles and quality standards as follows: host RNA cannot be detected by 0.8% agarose gel electrophoresis, protein ≤ 1 ng/µg plasmid, genomic DNA ≤ 0.002 µg/µg plasmid, endotoxin ≤ 10 EU/mg plasmid, and supercoiled plasmid DNA ≥ 90%, with a total purity of A_260_/A_280_ ≥ 1.75 [11–15]. The identification test was performed in accordance with the restriction map after restriction enzyme electrophoresis (Fig. 4).

One of the strengths of this study is that the combined purification method of PEG/MgCl_2_ and Triton X-114 effectively eliminated residual impurities from *E. coli* in the final supercoiled plasmid DNA product, thereby conforming to the international guidance for DNA vaccines [11–15]. The final purity of the supercoiled plasmid DNA obtained by combining PEG/MgCl_2_ precipitation with Triton X-114 was 94.98%, which was far higher than the standards of FDA and EAEM (90%). Notably, the efficiency of combining PEG/MgCl_2_ precipitation and Triton X-114 to remove endotoxins was higher than that of AEC and the commercial purification kit. Removal of endotoxin is always one of the bottlenecks in the purification process of plasmid DNA. This is because an endotoxin has a saclike structure, and its molecular weight, charge, and hydrophobicity are similar to those of plasmid DNA. Moreover, the molecular weight of an endotoxin ranges from hundreds of thousands to tens of millions, with a large number of negative charges. Therefore, reducing the content of endotoxin to a safe level using the molecular sieve and AC technologies currently used for the large-scale preparation of pharmaceutical plasmid DNA is challenging. In particular, *E. coli*, an engineered bacterium used to prepare DNA vaccines, contains a large amount of endotoxin. Usually, a concentration of 10% wet bacteria can produce tens of thousands of EU/mL of lipopolysaccharide (LPS), which is beyond the tolerance range of the human body. Because LPS has a strong heat source, a small amount can cause fever, blood circulation disorders, and even death from septic shock. Moreover, the presence of endotoxins can significantly affect the transfection efficiency of cells [32, 33]. Therefore, it is essential to remove endotoxin from the supercoiled plasmid DNA and ensure conformity to the relevant safety standards for the clinical application of plasmid DNA vaccines.

## Conclusions

To the best of our knowledge, this is the first demonstration that an innovative downstream process system using only a combination of PEG/MgCl_2_ precipitation with Triton X-114 that has been successfully developed for the large-scale production of the pcDNA-CCOL2A vaccine with high quality and yield. However, it remains uncertain whether this novel purification strategy and technical method can be extended to the separation and purification of other DNA vaccines. Currently, studies are underway to validate our methodology using a series of DNA vaccines for the treatment of type I diabetes, transplantation rejection, psoriasis, and other diseases.

## Data Availability

The datasets generated during the current study are available from the corresponding author upon reasonable request.

## Abbreviations

RA: Rheumatoid arthritis
FDA US: Food and Drug Administration
EAEM: Europe European Medicines Evaluation Agency
NMPA: China National Medical Products Administration
CIA: Collagen-induced arthritis
MTX: Methotrexate
LPS: Lipopolysaccharide
HPLC: High-performance liquid chromatography
LAL: Limulus amebocyte lysate
PEG: Polyethylene glycol

## References

[1] Onodera S, Ohshima S, Tohyama H, Yasuda K, Nishihira J, Iwakura Y (2007). A novel DNA vaccine targeting macrophage migration inhibitory factor protects joints from inflammation and destruction in murine models of arthritis. Arthritis Rheum.

[2] Xue H, Liang F, Liu N, Song X, Yuan F, Luo Y (2011). Potent antirheumatic activity of a new DNA vaccine targeted to B7–2/CD28 costimulatory signaling pathway in autoimmune arthritis. Hum Gene Ther.

[3] Shen Y, Chen J, Zhang X, Wu X, Xu Q (2007). Human TNF-alpha gene vaccination prevents collagen-induced arthritis in mice. Int Immunopharmacol.

[4] Ge PL, Ma LP, Wang W, Li Y, Zhao WM (2009). Inhibition of collagen-induced arthritis by DNA vaccines encoding TCR Vbeta5.2 and TCR Vbeta8.2. Chin Med J. (Engl).

[5] Song X, Liang F, Liu N, Luo Y, Xue H, Yuan F (2009). Construction and characterization of a novel DNA vaccine that is potent antigen-specific tolerizing therapy for experimental arthritis by increasing CD4^+^CD25^+^Treg cells and inducing Th1 to Th2 shift in both cells and cytokines. Vaccine.

[6] Song X, Liang F, Liu N, Luo Y, Yuan F, Xue H (2010). Therapeutic efficacy of experimental rheumatoid arthritis with low-dose methotrexate by increasing partially CD4+CD25+ Treg cells and inducing Th1 to Th2 shift in both cells and cytokines. Biomed Pharmacother.

[7] Zhao X, Long J, Liang F, Liu N, Sun Y, Xi Y (2019). Vaccination with a novel antigen-specific tolerizing DNA vaccine encoding CCOL2A1 protects rats from experimental rheumatoid arthritis. Hum Gene Ther.

[8] Zhao X, Long J, Liang F, Liu N, Sun Y, Xi Y (2021). Different protective efficacies of a novel antigen-specific DNA vaccine encoding chicken type II collagen via intramuscular, subcutaneous, and intravenous vaccination against experimental rheumatoid arthritis. Biomed Pharmacother.

[9] Long J, Zhao X, Yun S, Zhang Z, Jin J, Yu K (2015). Safety and immunogenicity of a novel therapeutic DNA vaccine encoding chicken type II collagen for rheumatoid arthritis in normal rats. Hum Vaccin Immunother.

[10] Zhao X, Long J, Liang F, Liu N, Sun Y, Xi Y (2019). Dynamic profiles, biodistribution and integration evaluation after intramuscular/intravenous delivery of a novel therapeutic DNA vaccine encoding chicken type II collagen for rheumatoid arthritis in vaccinated normal rodent. J Nanobiotechnology.

[11] Kutzler MA, Weiner DB (2008). DNA vaccines: ready for prime time?. Nat Rev Genet.

[12] Sousa A, Sousa A (2021). Biotechnological processes to obtain DNA vaccines. DNA vaccines.

[13] US Department of Health and Human Services. Guidance for industry: considerations for plasmid DNA vaccines for infectious disease indications; 2007. https://www.fda.gov/regulatory-information/search-fda-guidance-documents/considerations-plasmid-dna-vaccines-infectious-disease-indications. Accessed 13 Mar 2022.

[14] European Agency for the Evaluation of Medicinal Products. Note for guidance on the quality, preclinical, and clinical aspects of gene transfer medicinal products; 2001. https://www.ema.europa.eu/en/documents/scientific-guideline/note-guidance-quality-preclinical-clinical-aspects-gene-transfer-medicinal-products_en.pdf. Accessed 13 Mar 2022. London.

[15] Center for drug evaluation: technical Guidelines for preclinical studies of preventive DNA vaccines. NMPA. https://www.cde.org.cn/zdyz/domesticinfopage?zdyzIdCODE=bee5a01182004cca5c96f4d171cbdbbb. Accessed 12 May 2022.

[16] Xenopoulos A, Pattnaik P (2014). Production and purification of plasmid DNA vaccines: is there scope for further innovation?. Expert Rev Vaccines.

[17] Xi C, Liu N, Liang F, Guo S, Sun Y, Yang F (2006). Molecular cloning, characterization and localization of chicken type II procollagen gene. Gene.

[18] Long J, Zhao X, Liang F, Liu N, Sun Y, Xi Y (2018). Optimization of fermentation conditions for an Escherichia coli strain engineered using the response surface method to produce a novel therapeutic DNA vaccine for rheumatoid arthritis. J Biol Eng.

[19] Sambrook J, Rusell DW (2001). Molecular Cloning: A Laboratory Manual.

[20] Sauer ML, Kollars B, Geraets R, Sutton F (2008). Sequential CaCl_2_, polyethylene glycol precipitation for RNase-free plasmid DNA isolation. Anal Biochem.

[21] Ma R, Zhao J, Du HC, Tian S, Li LW (2012). Removing endotoxin from plasmid samples by Triton X-114 isothermal extraction. Anal Biochem.

[22] Khan M, Yan L, Lv B, Ji N, Shah S, Liu X (2020). The preparation of endotoxin-free genetically engineered murine B1 antisense RNA. Anal Biochem.

[23] Guerrero-Germán P, Prazeres DMF, Guzmán R, Montesinos-Cisneros RM, Tejeda-Mansir A (2009). Purification of plasmid DNA using tangential flow filtration and tandem anion-exchange membrane chromatography. Bioprocess Biosyst Eng.

[24] Sun B, Yu X, Yin Y, Liu X, Wu Y, Chen Y (2013). Large-scale purification of pharmaceutical-grade plasmid DNA using tangential flow filtration and multi-step chromatography. J Biosci Bioeng.

[25] Gonçalves GAL, Prather KL, Monteiro GA, Prazeres DMF (2014). Engineering of *Escherichia coli* strains for plasmid biopharmaceutical production: scale-up challenges. Vaccine.

[26] Long J, Zhao X, Yuan F, Liang F, Liu N, Yun S (2017). Genetic stability of an *Escherichia coli* strain engineered to produce a novel therapeutic DNA vaccine encoding chicken type II collagen for rheumatoid arthritis. Process Biochem.

[27] Stadler J, Lemmens R, Nyhammar T (2004). Plasmid DNA purification. J Gene Med.

[28] Lander RJ, Winters MA, Meacle FJ, Buckland BC, Lee AL (2002). Fractional precipitation of plasmid DNA from lysate by CTAB. Biotechnol Bioeng.

[29] Przybylowski M, Bartido S, Borquez-Ojeda O, Sadelain M, Rivière I (2007). Production of clinical-grade plasmid DNA for human phase I clinical trials and large animal clinical studies. Vaccine.

[30] Mourich DV, Munks MW, Murphy JC, Willson RC, Hill AB (2003). Spermine compaction is an efficient and economical method of producing vaccination-grade DNA. J Immunol Methods.

[31] Bo H, Wang J, Chen Q, Shen H, Wu F, Shao H (2013). Using a single hydrophobic-interaction chromatography to purify pharmaceutical-grade supercoiled plasmid DNA from other isoforms. Pharm Biol.

[32] Cotton M, Baker A, Saltik M, Wagner E, Buschle M (1994). Lipopolysaccharide is a frequent contaminant of plasmid DNA preparations and can be toxic to primary human cells in the presence of adenovirus. Gene Ther.

[33] Wicks IP, Howell ML, Hancock T, Kohsaka H, Olee T, Carson DA (1995). Bacterial lipopolysaccharide copurifies with plasmid DNA: implications for animal models and human gene therapy. Hum Gene Ther.

